# Profiling Immune Escape in Hodgkin’s and Diffuse large B-Cell Lymphomas Using the Transcriptome and Immunostaining

**DOI:** 10.3390/cancers10110415

**Published:** 2018-10-31

**Authors:** Sarah Péricart, Marie Tosolini, Pauline Gravelle, Cédric Rossi, Alexandra Traverse-Glehen, Nadia Amara, Camille Franchet, Elodie Martin, Christine Bezombes, Guy Laurent, Pierre Brousset, Jean-Jacques Fournié, Camille Laurent

**Affiliations:** 1 Centre de Recherches en Cancérologie de Toulouse, INSERM UMR1037, 31100 Toulouse, France; pericart.sarah@iuct-oncopole.fr (S.P.); marie.tosolini@inserm.fr (M.T.); pauline.gravelle@inserm.fr (P.G.); cedric.rossi66@gmail.com (C.R.); franchet.camille@iuct-oncopole.fr (C.F.); christine.bezombes@inserm.fr (C.B.); brousset.p@chu-toulouse.fr (P.B.); jean-jacques.fournie@inserm.fr (J.-J.F.); 2 Université Toulouse III Paul-Sabatier, 31330 Toulouse, France; amara.n@chu-toulouse.fr (N.A.); Laurent.Guy@iuct-oncopole.fr (G.L.); 3 Centre de Recherches en Cancérologie de Toulouse, ERL 5294 CNRS, 31100 Toulouse, France; 4 Laboratoire d’Excellence ‘TOUCAN’, 31059 Toulouse, France; 5 Programme Hospitalo-Universitaire en Cancérologie CAPTOR, 31059 Toulouse, France; 6 Institut Carnot Lymphome CALYM, 69495 Pierre-Bénite, France; 7 Departement de Pathologie, CHU Toulouse, Institut Universitaire du Cancer-Oncopole de Toulouse, 31059 CEDEX 09 Toulouse, France; 8 Hématologie Clinique, CHU Dijon, 21000 Dijon, France; 9 Hospices Civils de Lyon, Claude Bernard Lyon 1 University, INSERM 1052, 69002 Lyon, France; Alexandra.traverse-glehen@chu-lyon.fr; 10 Biostatistics Unit, Institut Claudius Regaud, IUCT-O, 31059 CEDEX 09 Toulouse, France; Martin.Elodie@iuct-oncopole.fr; 11 Departement d’Hematologie, CHU Toulouse, Institut Universitaire du Cancer-Oncopole de Toulouse, 31059 CEDEX 09 Toulouse, France

**Keywords:** lymphoma, immune escape, Hodgkin’s lymphoma, immune checkpoints, TIM-3, *datamining*

## Abstract

Therapeutic blockade of PD-1/PD-L1 shows promising results in Hodgkin’s lymphoma (HL) and in some diffuse large B-cell lymphoma (DLBCL) patients, but biomarkers predicting such responses are still lacking. To this end, we recently developed a transcriptional scoring of immune escape (IE) in cancer biopsies. Using this method in DLBCL, we identified four stages of IE correlated with overall survival, but whether Hodgkin’s lymphomas (HL) also display this partition was unknown. Thus, we explored the transcriptomic profiles of ~1000 HL and DLBCL using a comparative meta-analysis of their bulk microarrays. Relative to DLBCL, the HL co-clustered at the advanced stage of immune escape, displaying significant enrichment of both IE and T-cell activation genes. Analyses via transcriptome deconvolution and immunohistochemistry showed more CD3^+^ and CD4^+^ tumor-infiltrating lymphocytes (TILs) in HL than DLBCL. Both HL and non-GCB DLBCL shared a high abundance of infiltrating CD8^+^ T-cells, but HL had less CD68^+^CD163^+^ macrophages. The same cellular distribution of PD-1 and TIM-3 was observed in HL and DLBCL, though HL had more PD-L1 tumor cells and LAG-3 ME cells. This study illuminates the advanced stage of immune activation and escape in HL, consistent with the response to checkpoint blockade therapies for this type of lymphoma.

## 1. Introduction

In order to develop within immunocompetent hosts, it is imperative that tumors evolve several immune escape strategies [1,2,3]. Among them, the pro-tumor microenvironment (ME) and its interactions with cancer cells play a key role in promoting tumor cell growth and invasion. Lastly, the impairment of functional anti-tumor responses through the upregulation of the immune checkpoint (ICP) has further expanded the list of immune-escape mechanisms [4,5]. ICPs (among which the best known so far are CTLA-4, PD-1, LAG-3 and TIM-3) are lymphoid cell surface receptors which, by binding to their ligand, down-regulate immune responses. Upon massive overexpression of ICPs by tumor or ME cells, these pathways are subverted in cancer and impair anti-tumor immunity. The most widely studied ICP is currently PD-1/PD-L1, largely due to the impressive clinical efficacy of anti-PD-1 antibodies (e.g., nivolumab) in solid cancers and some lymphomas [6,7,8]. Recently, we and others have reported the overexpression of such ICPs in lymphomas by tumor cells and/or ME [5,9,10,11,12,13,14,15]. Expression of PD-1/PD-L1 in classical Hodgkin’s lymphoma (cHL) and in subtypes of diffuse large B-cell lymphoma patients (DLBCL) have been correlated with genetic alterations to the PD-L1 and PD-L2 locus of chromosome 9p24.1 (gains, amplifications, or fusions) [9,11,12,16,17]. These alterations are more frequently observed in Hodgkin Reed-Sternberg cells (HRS) than in DLBCL cells, and drive PD-L1,2 overexpression on tumor cells [12]. In addition, activation of the *JAK/STAT* pathway by inflammatory cytokines or activating mutations could also induce PD-L1 overexpression in these lymphoma [18,19,20]. Finally, EBV co-infection commonly seen in cHL can also upregulate PD-L1 on tumor cells via the EBV-encoded latent membrane protein (LMP)-1 that activates AP-1 (via *cJUN/JUN-B* components) or via *JAK/STAT* signaling pathways that activate the enhancer or promoter, respectively, of PD-L1 [21]. It is noteworthy that HRS cells, potentially targeted by PD-1 blockade, represent only 1% of cells from the cHL lymph node, while the rest contain an abundant and reactive cellular infiltrate. DLBCL tumors, in contrast, contain large numbers of malignant B-cells interspersed with far fewer non-neoplastic cells. However, the PD-1/PD-L1 axis is not exclusively expressed by tumor cells, but is also expressed by TILs or myeloid cells such as tumor-associated macrophages (TAM) or myeloid-derived suppressive cells (MDSC) [13,15,17,22]. Recently, multiplex immunofluorescence and digital image analysis have shown that the colocalization of PD-L1^+^ TAM with PD-L1^+^ HRS cells creates an immunoprotective niche that enhances locally augmented PD-1 signaling in cHL [13]. Despite the impressive clinical efficacy of anti-PD-1 antibodies (e.g., nivolumab) in cHL, and, to a lesser extent, in non-relapsed/refractory DLBCL patients [7,8,23,24], the lack of defined mechanisms of action for checkpoint blockade has impaired the prediction of therapeutic response and introduced a need to define the global status of IE for stratifying patients.

Recently, we have described a molecular signature based on upregulation of 33 immune-escape genes (IEGS signature) involved in immune escape [5,25], including gene encoding for ICPs (CTLA4, PDCD1, LAG3, HAVCR2, etc.), for their ligands (CD80, CD86, CD274, PDCD1LG2, LGALS9, etc.), for enzymes producing immunosuppressive metabolites (IDO1, ARG1, ENTPD1, etc.), and for immunosuppressive cytokines and chemokines (IL10, HGF, GDF15, etc.) (Supplemental Table S1). Although the immune escape strategies in lymphoma may vary between individuals, we have demonstrated that transcriptomic analysis of ~1500 B-NHL transcriptome microarrays consistently identifies significant upregulation of IEGS33 in follicular lymphoma, diffuse large B-cell lymphoma, mantle cell lymphoma, marginal zone lymphoma, hairy cell leukemia, and chronic lymphocytic leukemia [25]. Indeed, activation of immune effectors represents the ‘substrate’ of immune escape, and thus we scored each transcriptome from the 1500 samples for both ‘T-cell activation’ (44 genes such as *IL2*, *CD28*, *ZAP70*, *LCK*, (Supplemental Table S2)) and ‘IEGS33’. The resulting plots for both criteria revealed 4 different IE stages defined as follows: *Group 1* encompasses samples with low scores for both T-cell activation and IEGS33; *Group 2* has high “T-cell activation” and low IEGS33, *Group 3* has high “T-cell activation” and high IEGS33; while *Group 4* has low “T-cell activation” and high IEGS33 [25]. These four groups of samples also displayed different features such as mitotic activity, immune cell cytotoxicity, and immune infiltrates, which, when considered jointly, identified the following four stages of immune escape. *Stage 1*: non-immunogenic tumor; *Stage 2*: immunogenic, non-immuno-escaped tumor; *Stage 3*: Immunogenic, immune-escaped tumor, and *Stage 4*: Fully immune-escaped tumor [25]. Consistent with this staging, it was found that the stage 1 and 4 DLBCL patients IE had the worst clinical outcomes [25]. These findings also indicated that immune escape in DLBCL is a set of coordinated and redundant mechanisms, suggesting determinism and dynamics rather than an exclusive role from any single axis (such as PD-1/PD-L1,2). Here, we used this method to assess the heterogeneity of IE stages in HL and DLBCL patients, two diseases with different responses to ICPs blockade therapies.

## 2. Results

### 2.1. Molecular Profiling of Immune Escape in cHL and DLBCL

**Figure 1:**
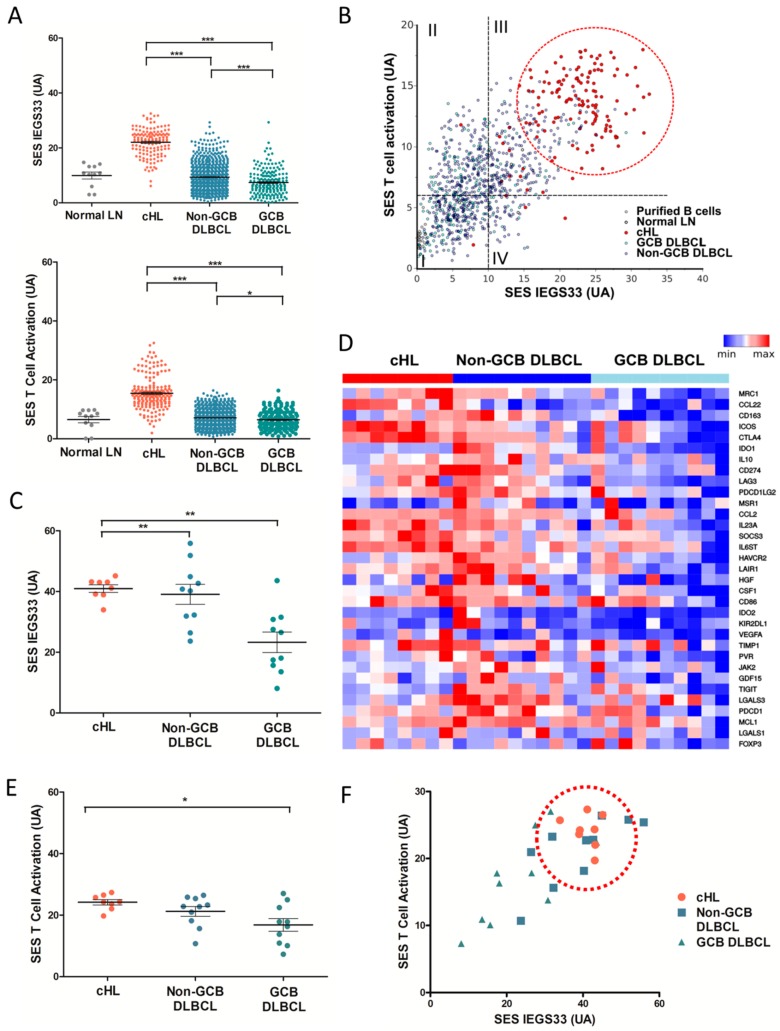
Molecular profiling of immune escape pathways in classical Hodgkin’s lymphoma and in diffuse large B-cell lymphoma. (**A**) Sample enrichment score (SES) for immune escape gene set IEGS33 (top) and for the T-cell activation gene set (bottom) in cHL, non-GCB DLBCL, GCB DLBCL, and reactive lymph node samples from public microarrays datamining analysis (*n* = 1061). (**B**) SES dot plots for IEGS33 versus T-cell activation for cHL, non-GCB DLBCL, GCB DLBCL, normal B-cell, and reactive lymph node samples from public microarrays datamining analysis (*n* = 1106), showing clustering of cHL samples at stage 3 of cancer IE (red circle), according to the 4 cancer IE stages, as previously defined [25] (cancer IE stage 1 (IEGS33^−^ T-cell activation^−^), cancer IE stage 2 (IEGS33^−^ T-cell activation^+^), cancer IE stage 3 (IEGS33^+^ T-cell activation^+^), cancer IE stage 4 (IEGS33^+^ T-cell activation^−^). (**C**) SES for IEGS33 in cHL, non-GCB DLBCL and GCB DLBCL samples from our private cohort (*n* = 28). (**D**) Heatmap of differential IE gene expression for cHL and DLBCL samples from our private cohort (*n* = 28). (**E**) SES for the T-cell activation gene set in cHL, non-GCB DLBCL, and GCB DLBCL samples from our private cohort (*n* = 28). (**F**) SES dot plots for IEGS33 versus T-cell activation for cHL, non-GCB DLBCL, GCB DLBCL samples from our private cohort (*n* = 28), showing clustering of cHL samples in stage 3 (red circle). (* *p* < 0.05, ** *p* ≤ 0.01, *** *p* ≤ 0.001).

We first measured and compared the enrichment score (SES) for IEGS33 using HGU 133 Plus 2.0 (Affymetrix) microarrays from an initial cohort of ~1000 cHL, DLBCL, and normal lymphoid tissue transcriptomes downloaded from the GEO data set (Methods). This publicly available cohort included *n* = 142 cHL, *n* = 908 DLBCL (including *n* = 720 non-GCB and 188 GCB subtypes), *n* = 11 non-cancer tissue controls taken from inflammatory lymphoid tissue biopsies, and *n* = 45 purified B-cells. As shown in Figure 1A, the SES for IEGS33 was significantly increased in cHL samples compared to GCB and non-GCB DLBCL subtypes (*p* ≤ 0.001), indicating the collective upregulation of these 33 genes in cHL compared to DLBCL, regardless of subtype. However, Figure 1A also depicts a significantly higher SES for IEGS33 in non-GCB DLBCL subtypes than in GCB subtypes (*p* ≤ 0.001). The enrichment of 44 genes involved in T-cell activation (downloaded from the MSigDB database) was scored, and demonstrated that cHL samples exhibited a higher SES for T-activation than non-GCB and GCB DLBCL (*p* ≤ 0.001) (Figure 1A). Since the activation of immune effectors represents the ‘substrate’ of immune escape, we then analyzed the relationship between these features by plotting the SES for IEGS33 versus the “T-cell activation” gene set. Figure 1B depicts that most (97%) cHL samples clustered together at immune escape stage 3, which corresponds to the advanced IE stage [25], whereas DLBCL samples were distributed among the four stages of cancer IE, with only 32% of non-GCB DLBCL assigned to stage 3.

We then assessed SES for IEGS and the T-cell activation gene set from HTA 2.0 microarrays (Affymetrix) of tumor biopsies from a second cohort of (*n* = 8) cHL and (*n* = 22) DLBCL patients treated in our institution, including 10 non-GCB DLBCL and 10 GCB DLBCL patients. Although the SES for random gene sets were generally higher from HTA microarrays than from HGU133 Plus 2.0 (data not shown), the SES for IEGS33 were higher in all cHL samples than in GCB DLBCL (*p* ≤ 0.01), but at the same level as in the non-GCB DLBCL samples (Figure 1C). Consistent with our previous results, the IEGS33 signature was significantly enriched in non-GCB DLBCL compared to GCB DLBCL (*p* ≤ 0.01) transcriptomes. In addition, the genes encoding for factors promoting tolerogenic ME or T-cell exhaustion were upregulated in cHL and non-GCB DLBCL compared to GCB DLBCL. These genes included CCL22, CTLA4, ICOS, IDO1, CD274, IL10, LAG3, PDCD1LG2, CCL2, SOCS3, IL6ST, HAVCR2, LAIR1, HGF, CSF1, CD86, IDO2, KIR2DL1, VEGFA, TIMP1, IL23A, PVR, MSR1, and JAK2 (*p* < 0.05). This differential gene expression pattern led to a clear-cut clustering between cHL/non-GCB DLBCL and GCB DLBCL samples based on IEGS33 gene expression levels (Figure 1D). Figure 1E shows that cHL displayed a higher SES for the T-activation gene set than GCB DLBCL (*p* < 0.05) and a tendency for higher SES than non-GCB DLBCL (*ns*). When plotting the SES for IEGS33 versus the “T-cell activation” gene set, all cHL samples also clustered together at stage 3, consistent with our previous results (Figure 1F). Taken in sum, these results highlighted the advanced IE signature of cHL.

### 2.2. Distribution of Immune Cells in cHL and DLBCL Tissue Samples

**Figure 2:**
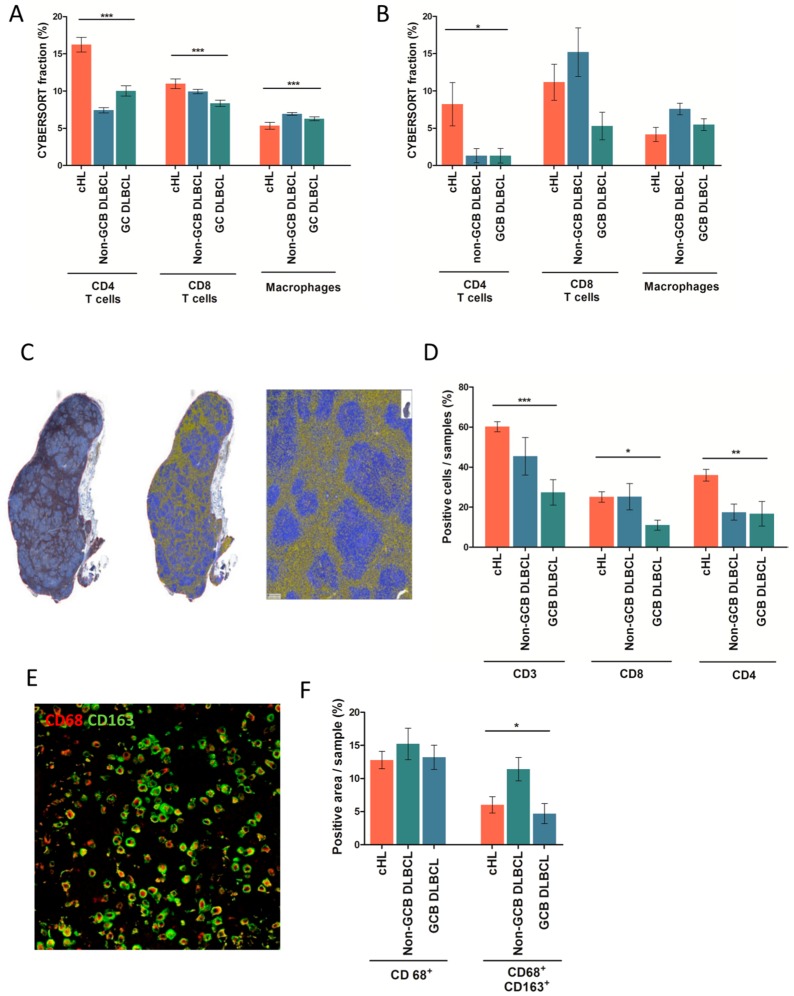
Immune subpopulation quantification based on microarray data and immunohistochemical studies in classical Hodgkin’s lymphoma and in diffuse large B-cell lymphoma. (**A**) T-cell and macrophage proportion (%) measured using the CIBERSORT deconvolution algorithm across cHL, non-GCB, and GCB DLBCL of public microarray data (*n* = ~1000) (statistical analysis performed using a one-way Anova test). (**B**) T-cell and macrophage proportion (%) measured using the CIBERSORT deconvolution algorithm in cHL, non-GCB, and GCB DLBCL microarray data from our own cohort (*n* = 28) (statistical analysis performed using a one-way Anova test). (**C**) Snapshot of automatic image analysis of CD3 staining using Definiens, Tissue Studio software. Example of IHC anti-CD3 on cHL lymph node (left panel; original magnification ×10). Example of quantification of CD3^+^ cells (yellow) and CD3^−^ cells (blue) (middle panel; original magnification ×10 and right panel; original magnification ×150). (**D**) Scoring based on IHC analysis of CD3^+^ T-cells, CD4^+^ T-cells, and CD8^+^ T-cells in cHL, non-GCB DLBCL, and GCB DLBCL samples from our cohort (*n* = 28) (statistical analysis performed using a one-way Anova test). (**E**) Representative staining of CD68+ (red) and CD163+ (green) macrophages in cHL lymph node (original magnification ×200) (statistical analysis as performed using a one-way Anova test). (**F**) Scoring of fluorescence intensity of CD68 and CD163 staining in cHL, non-GCB DLBCL, and GCB DLBCL samples from our cohort (*n* = 28) (statistical analysis was performed using a one-way Anova test). (* *p* < 0.05, ** *p* ≤ 0.01, *** *p* ≤ 0.001).

Since cHL and non-GCB DLBCL samples displayed the high score of immune cell exhaustion, we wondered whether they had similar patterns of leukocyte infiltration. To address this question, we used the CIBERSORT deconvolution algorithm [25] with modifications (Methods) to compute the proportion of each of 14 leucocyte and non-hematopoietic cell types for each sample. As shown in Figure 2A, deconvolution of the first cohort of *n* = ~1000 public microarrays showed higher rates of CD4^+^ T lymphocytes in cHL than in DLBCL, regardless of subtypes (*p* < 0.0001). Moreover, cHL and non-GCB DLBCL samples were enriched in CD8^+^ T-cells compared to GCB DLBCL (*p* < 0.0008). The cHL and GCB DLBCL samples had fewer infiltrating macrophages than non-GCB DLBCL (*p* < 0.0001) These observations were confirmed in the second cohort from our institution, in which cHL samples were significantly enriched in CD4^+^ T-cells compared to DLBCL (*p* < 0.05) regardless of GCB/non-GCB subtype (Figure 2B). In addition, cHL and DLBCL showed a tendency to have distinct proportions of macrophages (*p* = 0.06) and CD8^+^ T-cells (*p* = 0.07) (Figure 2B).

To further assess the immune architecture of cHL and DLBCL, we investigated the cellular composition of immune cell infiltrates using IHC and immunofluorescence analyses. We first calculated CD3^+^ T-cell infiltration on (*n* = 38) cHL and (*n* = 22) DLBCL FFPE samples using whole slide imaging combined with automated analysis for quantification (Figure 2C). This analysis showed that cHL comprise a significantly higher proportion of CD3^+^ T-cells (60% of total cells) than non-GCB or GCB DLBCL (45% and 27% of total cells, respectively) (*p* < 0.0004) (Figure 2D). The cHL samples were also characterized by a high abundance of CD4^+^ T-cells in their ME, accounting for a median 35% of all cells. In line with our previous molecular quantification (Figure 2A,B), the percentage of CD4^+^ T-cell in cHL was higher than in DLBCL, in which CD4^+^ T-cells accounted for only 18% (non-GCB DLBCL) and 17% (GCB DLBCL) of total cells (*p =* 0.002), respectively (Figure 2D). In addition, and in line with our previous molecular data, the samples of cHL and non-GCB DLBCL had a higher content of CD8^+^ T-cells than GCB DLBCL (25% of total cells versus 11% of total cells, *p* = 0.01) (Figure 2D).

We then calculated macrophage infiltration using multiplexed immunofluorescence analyses for CD68 and CD163 staining (Figure 2E). Consistent with the deconvolutions, the CD68^+^CD163^+^ macrophages were twice as abundant in non-GCB DLBCL (10% of tissue area) than in cHL (6% of tissue area) and GCB DLBCL samples (5% of tissue area) (*p* = 0.02) (Figure 1F). In contrast, the percentage of CD68^+^ macrophages did not vary significantly between cHL and DLBCL, regardless of subtype (15% in non-GCB, 13% in GCB, and 13% in cHL, *p* = 0.7) (Figure 1F). In sum, these IHC/IF results confirmed the leukocyte infiltration patterns quantified by CIBERSORT, namely that cHL contain more CD3^+^ and CD4^+^ T-cell infiltrates than DLBCL, yet share high rates of CD8^+^ T-cells with non-GCB DLBCL and low rates of CD68^+^CD163^+^ TAM with GCB DLBCL.

### 2.3. Distribution of Inhibitory Immune Checkpoint in HL and DLBCL Samples

**Figure 3:**
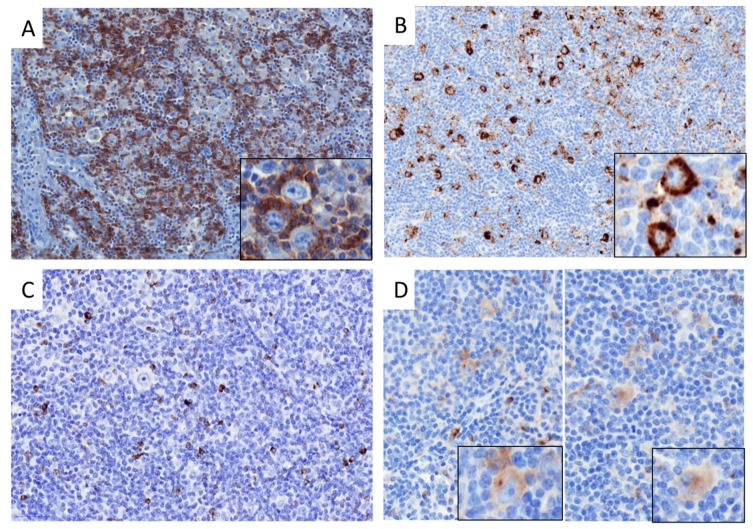
Immune checkpoint staining in classical Hodgkin’s lymphoma and in diffuse large B-cell lymphoma. (**A**) Representative staining of PD-1 in cHL sample showing PD-1^+^ small lymphocytes around tumor cells (magnification ×200, insert: ×400). (**B**) Representative staining of PD-L1 in cHL sample showing PD-L1^+^ immune cells and PD-L1^+^ HRS (magnification ×200, insert: ×400). (**C**) Representative staining of LAG-3 in cHL sample showing LAG-3^+^ small lymphocytes (magnification ×200). (**D**) Representative staining of TIM-3 in cHL sample showing TIM-3^+^ immune cells and TIM-3^+^ HRS (magnification ×200, insert: ×400).

**Figure 4:**
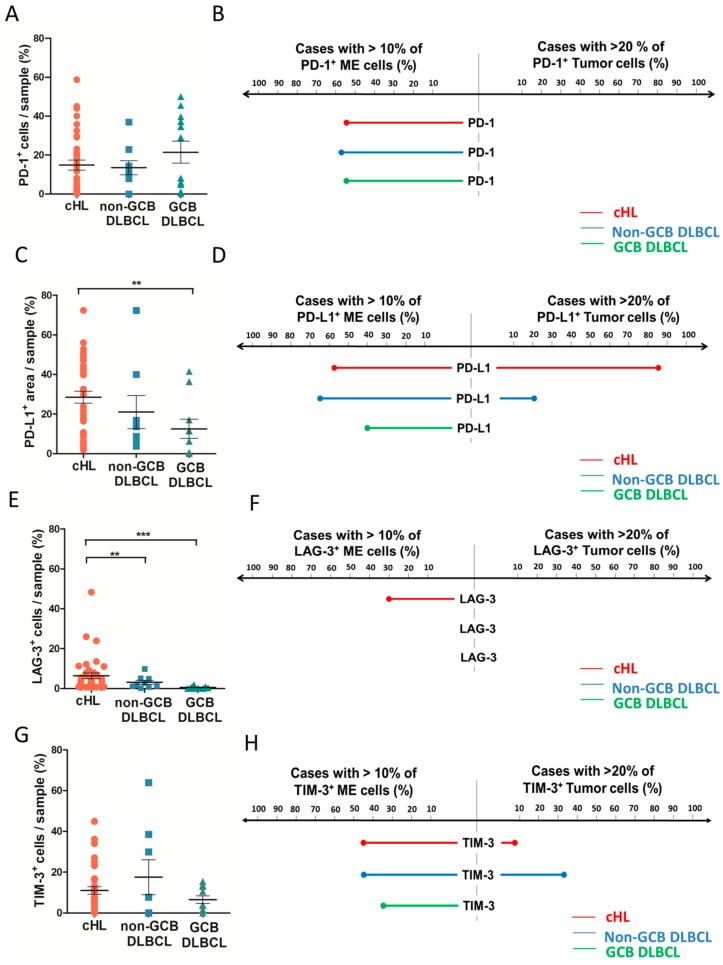
Quantification and cellular distribution of immune checkpoint expression in classical Hodgkin’s lymphoma and in diffuse large B-cell lymphoma from our cohort, by immunohistochemical studies. (**A**) Scoring of total PD-1^+^ cells/sample in cHL, non-GCB DLBCL and GCB DLBCL samples based on automated analysis. (**B**) Cellular distribution of PD-1 staining between tumor compartment (right) and micro-environment compartment (left) for cHL (red), non-GCB DLBCL (blue), and GCB DLBCL (green), with cutoff at >10% of ME immune cells and >20% of tumor cells. (**C**) Quantification of total PD-L1^+^ area/sample (% of area positive) in cHL, non-GCB DLBCL, and GCB DLBCL samples based on automated analysis. (**D**) Cellular distribution of PD-L1 staining between tumor compartment (right) and micro-environment compartment (left) for cHL (red), non-GCB DLBCL (blue), and GCB DLBCL (green) with cutoff at >10% of ME immune cells and >20% of tumor cells. (**E**) Quantification of total LAG-3^+^ cells/sample (% of cells positive) in cHL, non-GCB DLBCL, and GCB DLBCL samples based on automated analysis. (**F**) Cellular distribution of LAG-3 staining between tumor compartment (right) and micro-environment compartment (left) for cHL (red), non-GCB DLBCL (blue), and GCB DLBCL (green) with cutoff at >10% of ME immune cells and >20% of tumor cells. (**G**) Quantification of total TIM-3^+^ cells/sample (% of cells positive) in cHL, non-GCB DLBCL, and GCB DLBCL samples based on automated analysis. (**H**) Cellular distribution of TIM-3 staining between tumor compartment (right) and micro-environment compartment (left) for cHL (red), non-GCB DLBCL (blue), and GCB DLBCL (green) with cutoff at >10% of ME immune cells and >20% of tumor cells. (* *p* < 0.05, ** *p* ≤ 0.01, *** *p* ≤ 0.001).

The above analyses indicated that despite distinct patterns of immune infiltration, both cHL and non-GCB DLBCL upregulate the transcription of most IEGS genes, raising the question of which cells—malignant or microenvironmental—express these genes. Therefore, we investigated the protein expression and cellular distribution of inhibitory immune checkpoints and their ligands in our cHL and DLBCL cohort. As shown in Figure 3A and Figure 4A, the proportion of total PD-1^+^ cells was not significantly different between cHL and DLBCL, with an average of 16% PD-1^+^ cells. In contrast and in accordance with published data [5], the proportion of total cells expressing PD-L1 was higher in cHL (29% of total cells) and non-GCB (23% of total cells) than in GCB DLBCL (13% of total cells) (*p* = 0.06) (Figure 3B and Figure 4C). The proportion of total LAG-3^+^ cells was twice higher in cHL (6% of total cells) than in non-GCB (3% of total cells) and GCB DLBCL (1% of total cells) (*p* ≤ 0.0001) (Figure 3C and Figure 4E). The percentage of TIM-3^+^ cells was not significantly different between cHL and DLBCL, accounting for a median of 11%, 16% and 7% of cHL, non-GCB DLBCL, and GCB DLBCL tumors, respectively (*p* = 0.5) (Figure 3D and Figure 4G).

To determine the specific cellular distribution of ICP expression in lymphoma samples, we evaluated the staining of PD-1, PD-L1, LAG-3, and TIM-3 in the ME and in tumor cells with a cutoff value of 10% for ME cells and 20% for tumor cells. As shown in Figure 4B, PD-1 was expressed exclusively by the ME cells in all lymphoma cases. In contrast, PD-L1 was expressed in both ME and tumor cells of cHL and non-GCB DLBCL, but only in the ME cells of GCB DLBCL (Figure 4D). As expected, PD-L1 was expressed more frequently by tumor cells in cHL than in non-GCB DLBCL (86% and 22% of cases respectively, *p ≤* 0.0001). LAG-3 staining was observed exclusively in the ME cells of all cHL and DLBCL cases (Figure 4F). Finally, TIM-3 was expressed by both non-tumor and tumor cells in 10% of cHL and 35% of non-GCB DLBCL cases, whereas TIM-3 was expressed exclusively in the ME of GCB DLBCL and never detected in GCB DLBCL tumor cells (Figure 4H). Taken in sum, these results demonstrated that cHL and DLBCL overexpress most inhibitory ICPs in their ME and tumor cells. Of note is the fact that LAG-3 is significantly overexpressed in cHL but barely expressed in DLBCL, while PD-L1 is overexpressed by cHL and non-GCB DLBCL, suggesting that these tumor cells are prone to modulating immune activation responses.

### 2.4. Clinical Correlates of Immune Status

**Figure 5:**
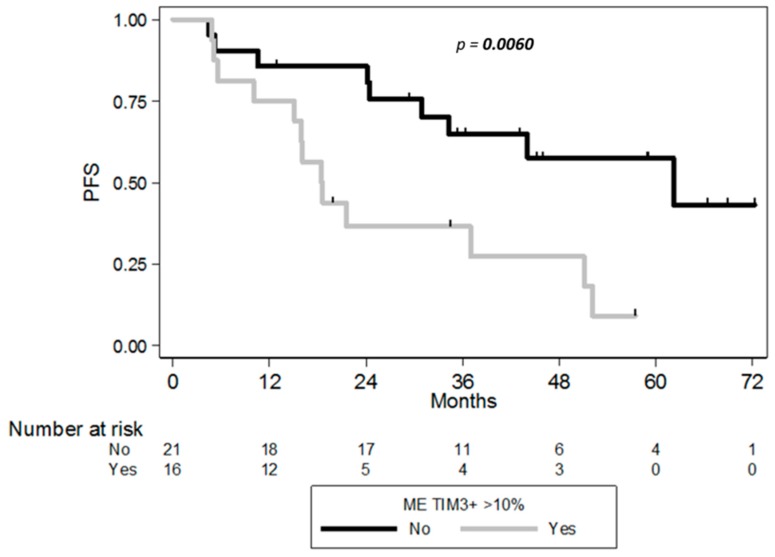
Progression Free Survival (PFS) curves according to the percentage of TIM-3^+^ ME cells of cHL patients (*n* = 37), with cutoff of 10% of ME immune cells positive (*p* = 0.006).

The prognostic values of ICP expression in cHL and DLBCL were assessed using the Kaplan–Meier method and log-rank test in order to outline any association of LAG-3, PD-1/PD-L1, and TIM-3 expression with clinical follow-up information. Clinical outcome was available in 37/38 cHL and in 22/22 DLBCL patients. The patients were divided into high/low protein expression groups according to the cutoff value of 10% using whole slide imaging. In univariate analysis, high TIM-3 expression in non-tumor components was associated with shortened progression-free survival (PFS) of cHL patients (hazard ratio 3.28, 95% CI 1.34–8.00, log rank *p*-value = 0.006). This suggested that high expression of TIM-3 on tumor infiltrating cells was associated with poor prognosis in cHL (Figure 5). Moreover, global staining of TIM-3 quantified by automated analysis (including staining in HRS cells) has no significant association with shorter PFS (*p* = 0.16). PD-1, PD-L1, or LAG-3 expression alone was also not associated with shortened PFS in cHL patients (*p* = 0.5; *p* = 0.7; *p* = 0.6, respectively). Even at the protein level, expression of each ICP (considered in isolation) did not correlate with the clinical outcome of DLBCL patients from our cohort, regardless of cellular distribution of the IHC staining (data not shown). However, in the 200 clinically annotated samples (of the ~1000 DLBCL and cHL samples), the clinical outcome of DLBCL patients was strikingly different according to the 4 groups defined by IE stages. In this public cohort, however, no clinical annotation was available for the cHL patients, whose samples were assigned solely to stage 3 IE. In accordance with previous data (ref), the 5-year OS of DLBCL patients decreased from stage 2 (70%) to stage 3 (48%), stage 1 (44%), and stage 4 (38%) (log Rank *p* = 0.0006) (Supplemental Figure S1).

## 3. Discussion

Given the impressive therapeutic response to PD-1 antibodies recently reported in HL [7,8], an overview of IE pathways and characterization of immune cells distribution in HL is helpful for understanding the process of immune restoration and thus predicting immune therapy strategy responses. This study combining transcriptome analysis and multi-parametric IHC has revealed that non-GCB DLBCL, GCB DLBCL, and cHL tumors display distinct immune escape strategies, notably through upregulation of several immune checkpoints by tumor cells and infiltrating immune cells. cHL and non-GC DLBCL are characterized by a high expression of immune escape genes and an overexpression of ICPs by both tumor cells and ME cells, indicating that these lymphoma subtypes are prone to modulating immune response. As previously established, the overexpression of PD-L1 observed in cHL and non-GCB DLBCL is associated with an adverse prognosis in HL and DLBCL patients [9,12,15], suggesting that the PD-1/PD-L1 axis plays a key role in IE strategies for these lymphomas. However, response rates to blockading the PD-1/PD-L1 axis in relapsed/refractory DLBCL patients are not as high as in HL [23,24]. This suggests that blocking PD-1/PD-L1 might suffice to restore anti-tumor immune response in HL patients, but not in DLBCL patients. The different response rates to PD-1 antibodies in these lymphomas could be related to a distinct capacity of effector T-cells to lyse tumor cell upon immune therapy. The high level of T-cell activation gene expression in cHL observed in this study implies that these tumors contain a pre-existing pool of tumor-specific T-cells, which could be able to reinvigorate immune responses upon immunotherapy. In certain solid tumors, such as melanoma, PD-1 blockade has been linked effectively to CD8^+^ cytotoxic T-cell reactivation [26,27]. However, the frequent antigen losses and alterations of the HLA class-I antigen presentation on HRS cells suggests that in cHL, PD-1 blockade might also involve a non-CD8 T-cell-mediated mechanism [28,29]. The potential role of CD4^+^ T-cells in immune responses to HL has been suggested by the physical proximity of PD-L1^+^ HRS cells to PD1^+^ CD4^+^ cells [13]. Likewise, the association between MHC II expression and the predictive response to anti-PD-1 therapy further support the key role of CD4 T-cells [30]. In line with this data, the present study shows a different composition of immune cells infiltrating tumors in cHL and DLBCL. Notably, cHL display a higher abundance of CD4^+^ TILs than DLBCL, consistent with literature [31,32,33], and further supporting the critical role of CD4^+^ T-cells in anti-tumor immunity in HL and as target for immunotherapy.

In contrast to a previous study [22] assessing CD163 gene expression in HL and DLBCL regardless of subtypes, here, both CIBERSORT and IHC analysis substantiated the lower infiltration of CD68^+^CD163^+^ macrophages in cHL compared to non-GCB DLBCL. The discrepancy could rely on distinct means to characterize the monocyte/macrophage subpopulations [34] in both studies, but also on the different macrophage abundance in tumors from non-GCB and GCB DLBCL subtypes (a distinction absent from the earlier report). Our results also highlight the key role of M2 monocytes/macrophages in progression of non-GCB DLBCL. This was evidenced by the upregulated expression of IEGS33 genes such as those encoding for the CCL2, CCL22, and CSF1 chemokines that promote immunosuppressive cells (e.g., M2 macrophages or TAM) in the lymphoma microenvironment as well as the upregulation of immunosuppressive molecule genes (such as indole 2,3-dioxygenase (IDO) which induces T-cell inhibition).

Further investigations with methods such as CyTOF and multiplexed imaging will allow us to evaluate monocyte/macrophages in HL immune responses.

Nevertheless, the different composition of tumor-infiltrating immune cells in HL, non-GCB DLBCL, and GCB DLBCL might account for the differing impact of ICP blockade in these patients. Alternatively, the different responses to PD-1 antibodies in DLBCL and HL patients could also be related to additional IE mechanisms such as the expression of other ICPs. As reported before, LAG3 and TIM3 have high expression in HL microenvironments—particularly in T-cells [35]. This study also showed that LAG-3 is expressed more often in cHL samples than in DLBCL, regardless of subtype. Although the exact mechanism of action is still unknown, LAG-3 is a negative regulator in CD4 and CD8 T-cell expansion, frequently co-expressed with PD-1 in TILs [36,37]. Hence, the combination of LAG-3 and PD-1 blockade increases the anticancer activity of TILs [37]. Furthermore, the expression of TIM-3 was also observed in TILs in cancer including lymphoma [14,38,39,40,41,42] and its interaction with Galectin ligands inhibits T-cell function [43,44]. The efficacy of TIM-3 blockade in preclinical murine models of melanoma positions TIM-3 as a target suitable for cancer immunotherapy [45]. In contrast to LAG-3, cHL and DLBCL exhibit the same level of TIM-3 protein, while both cHL and non-GCB DLBCL tumor cells express TIM-3. In several solid cancers, TIM-3 expression by TILs is associated with the least favorable prognosis [38,39,40,41,42]. Likewise, TIM-3 staining on immune cells from cHL was associated with the leave favorable prognosis in our experiments. These results could help identify HL patients who might benefit from ICP blockade, either alone or in conjunction with other treatments, as suggested by El Halabi et al. [35]. Unfortunately, we did not find any correlation between clinical outcome and the expression of ICPs in DLBCL patients, probably due to the limited cohort studied. However, consistent with the previous study [25], we found that both IE and T-cell activation gene enrichment in transcriptomes delineate four IE stages associated with overall survival in DLBCL patients. Interestingly, cHL clustering at the advanced stage of immune escape reveals that cHL are not immune deserts, but rather highly immunogenic tumors, accounting for their high rates of therapeutic responses to PD-1-blockade, most notably when compared to DLBCL.

## 4. Materials and Methods

### 4.1. Patients

The study cohort consisted of primary diagnosis of cHL (*n* = 38) and DLBCL (*n* = 22) patients who were diagnosed in the Department of Hematology, IUCT France and in the Department of Hematology, Lyon between 2006 and 2015 according to the WHO classification. Of note is the fact that all DLBCL patients were enrolled in the AMARE program [46]. All clinical data was available for 37/38 cHL patients and 22/22 DLBCL patients and is described in Supplemental Table S3.

The study was conducted in accordance with the Helsinki Declaration and performed with written informed patient consent. All HL and DLBCL FFPE samples are stored at the IUCT in the CRB biobank collection (BB-0033-00014) which has been declared to the Ministry of Higher Education and Research (DC-2008-463) and obtained a transfer agreement (AC-2008-820) after receiving approval from the ethical committee (CPP).

### 4.2. Microarray Analysis

The public GEO repository [47] for gene expression profiles of cHL and DLBCL, obtained with the Affymetrix HG U133 plus 2.0 microarray, was used in conjunction with 12 downloaded datasets (GSE10524, GSE10846, GSE11318, GSE12195, GSE12453, GSE17189, GSE17920, GSE2109, GSE31312, GSE34171, GSE3526, and GSE7307). These data sets were checked for compatibility, and whenever appropriate, these were assembled, RMA-normalized, and collapsed to ~20,000 HUGO protein-encoding genes as previously described [25], yielding a meta-series of (*n* = 1106) transcriptomes from lymphoma and healthy sample controls. These encompassed cHL (*n* = 142), DLBCL (*n* = 908), as well as normal samples of lymph node (*n* = 11) and (*n* = 45) purified B-cell controls. We also download gene sets from Gene Ontology [48], KEGG (http://www.genome.jp/kegg/pathway.html), and the Molecular Signatures Data base (MSigDB 3.0) [49]. The SING14 gene set was modified from [50] and is provided (Supplemental Table S4). Each transcriptome was processed for robust statistical analysis and single sample gene set enrichment analysis were computed for various gene sets such as T-cell activation (MSigDB) and the immune escape-defining IEGS33 gene set as previously described [5,25]. The list of IEGS33 and T cell activation genes is described in Supplemental Tables S1 and S2 respectively. The leukocyte composition of the cHL and DLBCL tumors was deconvoluted from their microarrays using the CIBERSORT algorithm as described previously [51], with some modifications [25].

Frozen samples of cHL (*n* = 8) and DLBCL (*n* = 20) from our institution were available and used for transcriptomic study. Frozen sections were cut from each of the cryopreserved samples and examined for adequacy of the materials before other studies. Total mRNA was extracted using an RNA Mini Kit (QIAGEN). cDNA was prepared from 500 pg RNA per sample and hybridized on GeneChip Human Gene ST 2.0 Affymetrix microarrays (Affymetrix UK Ltd., High Wycombe, UK), by the Lyon University genomic facility ProfileXpert-LCMT (Lyon, France) according to the manufacturer’s protocol. Data was logged on the NCBI Gene Expression Omnibus (GEO) website (http://www.ncbi.nlm.nih.gov/geo/) and is available as a GSE120124 dataset. Raw data was RMA–normalized and collapsed to unique protein-encoding genes (using the HUGO nomenclature). Then, the single sample gene set enrichment analysis for IEGS33 and T-cell activation gene sets and leukocyte composition by the CIBERSORT was performed as described previously for public datasets (see the beginning of Section 4.2).

### 4.3. Immunohistochemistry

For IHC studies, the 8 cHL samples with 30 additional cHL samples and the 20 DLBCL samples with 2 additional DLBCL samples were used for immunostaining. Four µm-thick sections of the formalin-fixed paraffin-embedded (FFPE) cHL and DLBCL lymph node whole tissues were stained with hematoxylin and eosin (HE, Sakura TissueTek, Sakura, Tokyo, Japan). Immunohistochemical stains for CD3 (2GV6; prediluted; Roche, Oro Valley, AZ, USA), CD4 (SP35; prediluted; Roche), CD8 (SP57; prediluted; Roche), PD-1 (NAT 105; prediluted; Roche), PD-L1 (SP162; prediluted; Roche), LAG-3 (polyclonal; 1:500; Novus Biologicals, Littleton, CO, USA) and TIM-3 (polyclonal;1:50; R&D, Minneapolis, MN, USA) were realized according to the manufacturer recommendation using Ventana Benchmark XT immunostainer (Ventana, Tucson, AZ, USA). The IHC slides were digitalized using Panoramic 250 Flash II digital microscopes (3DHISTECH, Budapest, Hungary). IHC staining was evaluated both via manual scoring and an automated method using image analysis software (Tissue Studio, Definiens, Munich, Germany) with substantial intra-class correlation coefficient (Supplemental Table S5).

To assess cell-of-origin (COO) groups of DLBCL cases by IHC, tissue were stained for CD10 (56C6; 1:100; Novocastra-Leica microsystems, Leica Biosystems Newcastle Ltd, Newcastle, UK), BCL6 (PG-B6p; 1:30; Dako, Carpinteria, CA, USA), IRF4/MUM1 (MUM1p; 1:25; Dako), and classified using the Hans algorithm [52]. Comparisons were made with COO assignment of DLBCL using the original GEP methods and published algorithm [53] (Supplemental Figure S2).

### 4.4. Immunofluorescence

Samples were pre-treated by microwave incubation in pH 9 (Dako Traget retrieval). Samples were then permeabilized with 0.1% saponin (in PBS 3% BSA/HEPES, 10% human serum), and stained ON at 4 °C with the following two primary antibodies: CD68 (PGM1; 1:100; Dako) and CD163 (10D6; 1:100; Novocastra). Primary Abs were then targeted by goat anti-mouse isotype specific Ab labelled with Alexa 488 and Alexa 594 for 2 h at RT. The samples were mounted in Fluorescence Mounting Medium^®^ (Dako) and examined using a Zeiss LSM 710 confocal microscope (Zeiss, Oberkochen, Germany). For each pair of antibodies used, standardized conditions for pinhole size, gain, and offset (brightness and contrast) were used for image capture. Images were analyzed using *ImageJ* software and quantification of stain was calculated as area of fluorescence on total tissue area for each sample.

### 4.5. Statistical Analyses

Comparisons of lymphoma subtypes concerning gene and protein expression were performed using the Student *t*-test or Mann–Whitney test (two-tailed, unpaired) according to normal or abnormal distribution of values, respectively. A one-way Anova test was also used to compare lymphoma groups for cellular abundance. *p*-values of <0.05 were considered statistically significant (* *p* < 0.05, ** *p* ≤ 0.01, *** *p* ≤ 0.001).

Progression free survival was defined as the time from the initiation of treatment to progression or death from any cause, and patients without an event were censored at the date of the last follow-up. Univariable survival analyses were conducted using the log rank test and Cox proportional hazards regression model. PFS rates were estimated using the Kaplan–Meier method with 95% confidence intervals (CI). Each case was classified as high or low expression based on percentage of total cells, micro-environment cells, or tumor cells expressing each marker, with cutoff based on median expression and data from the literature (10% for ME cells and 20% for tumor cells).

The intra class correlation coefficients were measured using two-way mixed-effects model-Absolute agreement. Tests were two-sided and *p*-values of <0.05 were considered significant. Statistical analyses were conducted using Stata^®^, version 13.

## 5. Conclusions

In conclusion, this study highlights an advanced stage of immune evasion occurring in all HL tumors, which accounts for the success of anti-PD-1 blockade in treating this disease. Thus, bioinformatics and transcriptomic data constitute essential tools for characterizing the immune status of tumors from lymphoma patients and determining those most eligible for ICP strategies, prior to using these tools for immune-monitoring during future clinical ICPs trials.

## Supplementary Materials

The following are available online at http://www.mdpi.com/2072-6694/10/11/415/s1, Figure S1: Overall survival (OS) curves according to the four stages of IE of DLBCL patients from public microarray data (*n* = 200) (log rank *p* = 0.0006), Figure S2: Classification of DLBCL subtypes based on the Hans algorithm [52] and on GEP [53] in our private cohort, Table S1: List of “IEGS33” immune escape gene set, Table S2: List of T cell activation gene set, Table S3: Table of clinical characteristics of patients from our private cohort, Table S4: SING14 matrix used for CIBERSORT deconvolution,, Table S5: Intra-class correlation coefficient between manual scoring and automated scoring for IHC: CD3, CD8, CD4, PD-1, PD-L1, TIM-3, and LAG-3.
