# Supplementary material for: Profiling Immune Escape in Hodgkin’s and Diffuse large B-Cell Lymphomas Using the Transcriptome and Immunostaining
Source: Cancers (Basel). 2018 Oct 31;10(11):415. doi: 10.3390/cancers10110415 (PMC6266061; doi:10.3390/cancers10110415)
Supplement: Supplementary file 1 [file cancers-10-00415-s001.zip › Figures_tables_supplemental_revised_proof/Figure S1.pptx]

## Slide 1
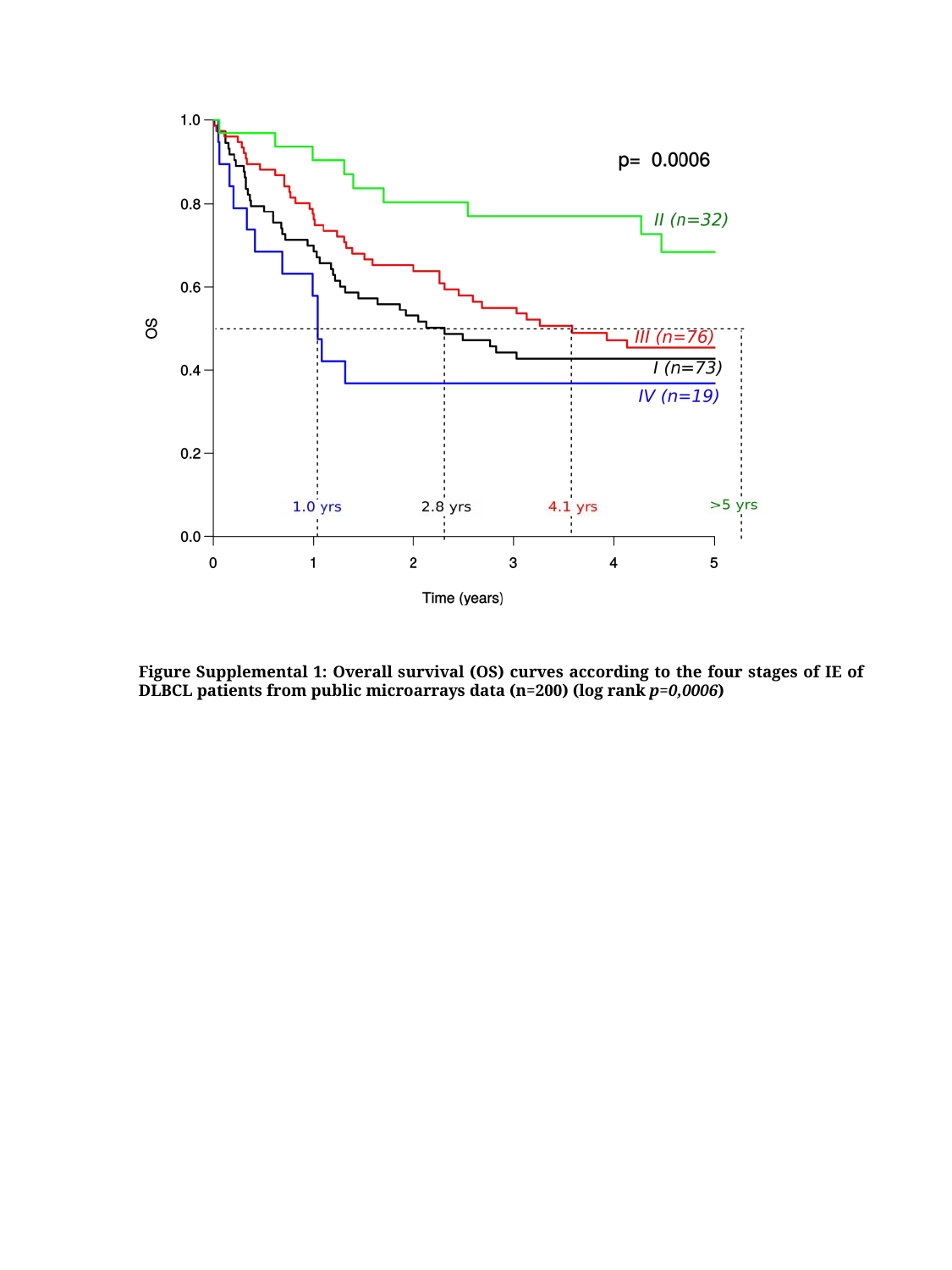

Figure Supplemental 1: Overall survival (OS) curves according to the four stages of IE of DLBCL patients from public microarrays data (n=200) (log rank p=0,0006)
