# Supplementary material for: Profiling Immune Escape in Hodgkin’s and Diffuse large B-Cell Lymphomas Using the Transcriptome and Immunostaining
Source: Cancers (Basel). 2018 Oct 31;10(11):415. doi: 10.3390/cancers10110415 (PMC6266061; doi:10.3390/cancers10110415)
Supplement: Supplementary file 1 [file cancers-10-00415-s001.zip › Figures_tables_supplemental_revised_proof/figure S2.pptx]

## Slide 1
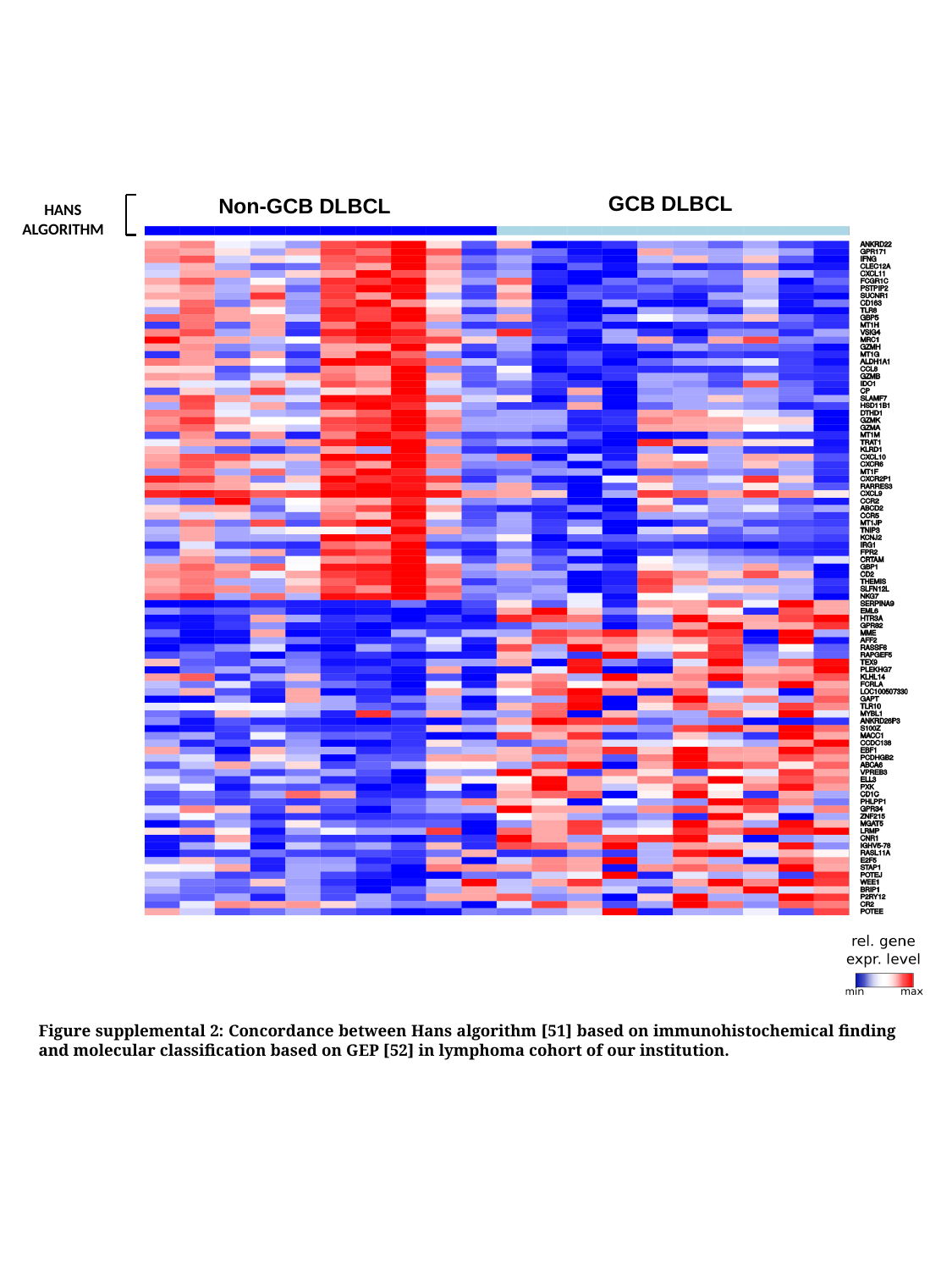

GCB DLBCL
Non-GCB DLBCL
HANS ALGORITHM
Figure supplemental 2: Concordance between Hans algorithm [51] based on immunohistochemical finding and molecular classification based on GEP [52] in lymphoma cohort of our institution.
