# Supplementary material for: Profiling Immune Escape in Hodgkin’s and Diffuse large B-Cell Lymphomas Using the Transcriptome and Immunostaining
Source: Cancers (Basel). 2018 Oct 31;10(11):415. doi: 10.3390/cancers10110415 (PMC6266061; doi:10.3390/cancers10110415)
Supplement: Supplementary file 1 [file cancers-10-00415-s001.zip › cancers-368469-Supplement-final/cancers-368469-Figures 1 and 2 and Table S5.pdf]

cancers

*Supplementary Figures*

# Profiling Immune Escape in Hodgkin's and Diffuse large B-Cell Lymphomas Using the Transcriptome and Immunostaining

**Sarah Pericart, Marie Tosolini, Pauline Gravelle, Cédric Rossi, Alexandra Traverse-Glehen, Nadia Amara, Camille Franchet, Elodie Martin, Christine Bezombes, Guy Laurent, Pierre Brousset, Jean-Jacques Fournié and Camille Laurent**

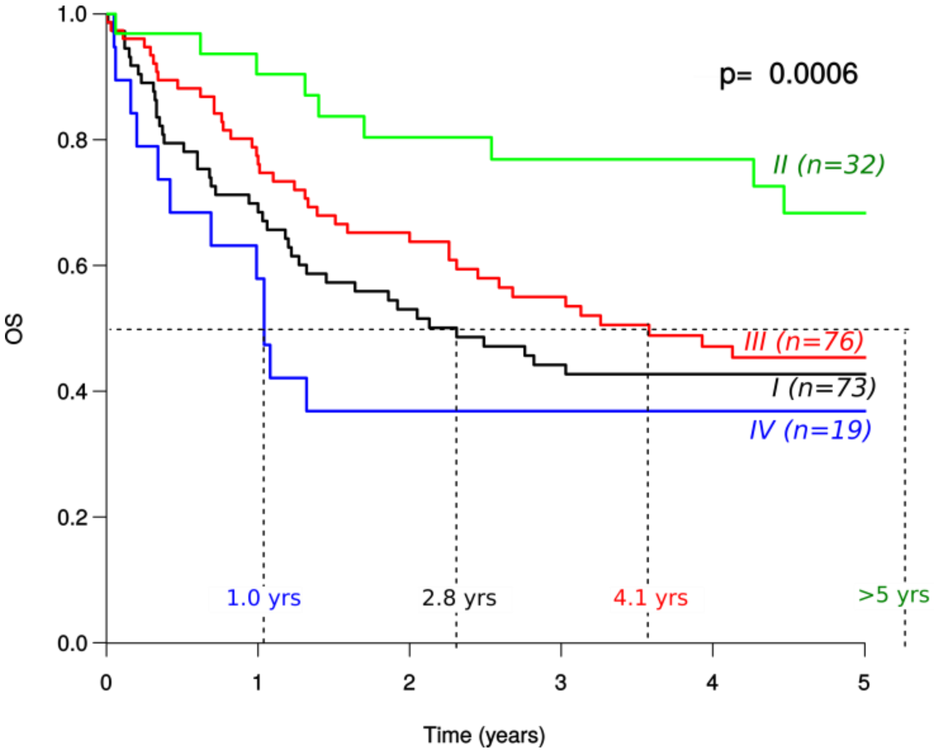


**Figure S1**. Overall survival (OS) curves according to the four stages of IE of DLBCL patients from public microarrays data (n = 200) (log rank $p = 0.0006$).

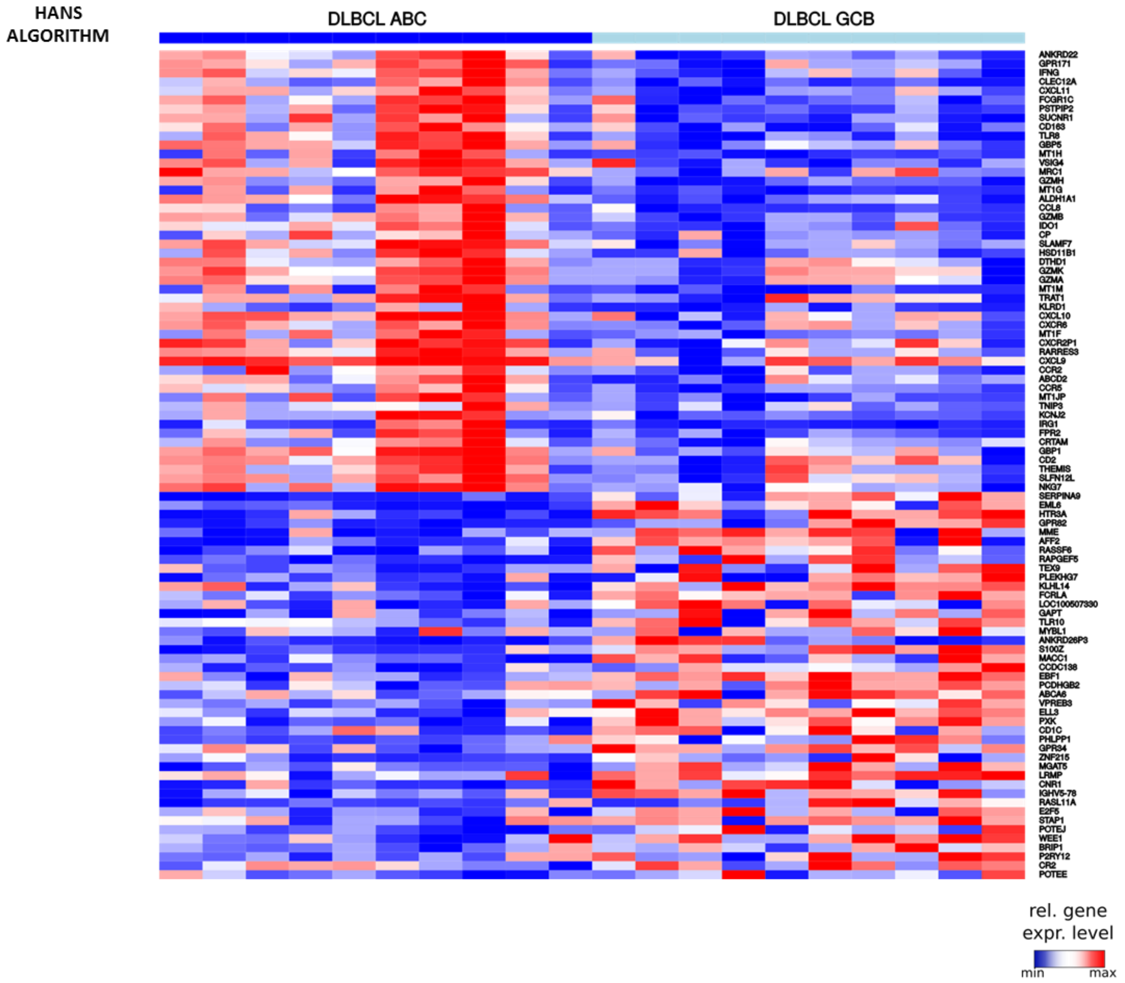


**Figure S2.** Concordance between Hans algorithm [51] based on immunohistochemical finding and molecular classification based on GEP [52] in lymphoma cohort of our institution.

**Table S5.** Intra-class correlation coefficient between manual scoring and automated scoring for IHC: CD3, CD8, CD4, PD-1, PD-L1, TIM-3 and LAG-3.

| IHC | ICC | [95% CI] |
|---|---|---|
| CD3 | 0.75 | [0.52 ; 0.87] |
| CD8 | 0.81 | [0.61 ; 0.90] |
| CD4 | 0.86 | [0.75 ; 0.92] |
| PD-1 | 0.70 | [0.49 ; 0.84] |
| PD-L1 | 0.87 | [0.68 ; 0.94] |
| TIM3 | 0.72 | [0.52 ; 0.85] |
| LAG3 | 0.89 | [0.74 ; 0.95] |
